# Prevalence of a carbapenem-resistance gene (KPC), vancomycin-resistance genes (*van* A/B) and a methicillin-resistance gene (*mec*A) in hospital and municipal sewage in a southwestern province of Saudi Arabia

**DOI:** 10.1186/s13104-018-3167-2

**Published:** 2018-01-15

**Authors:** Vinod Kumar Basode, Ahmed Abdulhaq, Mohammed Uthman A. Alamoudi, Hassan Mohammad Tohari, Waleed Ali Quhal, Aymen Mohammed Madkhali, Yahya Hasan Hobani, Almonther Abdullah Hershan

**Affiliations:** 1 Unit of Medical Microbiology, Department of Medical Lab Technology, Faculty of Applied Medical Sciences, Jazan University, Jazan, Saudi Arabia; 2 Deanship of Scientific Affairs and Research, Jazan University, Jazan, Saudi Arabia; 3 Unit of Medical Molecular Biology and Genetics, Department of Medical Lab Technology, Faculty of Applied Medical Sciences, Jazan University, Jazan, Saudi Arabia; 4 Department of Medical Microbiology and Parasitology, University of Jeddah, Jazan, Saudi Arabia

**Keywords:** Vancomycin-resistance genes, Carbapenem-resistance gene, Methicillin resistance gene, Sewage

## Abstract

**Objective:**

According to the World Health Organization, the increasing antibiotic resistance of pathogens is one of the most important threats to human health. Prevalence of a carbapenem-resistance gene (KPC), vancomycin-resistance genes (*van* A/B) and a methicillin-resistance gene (*mec*A) in hospital and municipal sewages will be potential threat to public health.

**Results:**

Vancomycin-resistance genes were detected in the sewage of community tank-II, sewage tank of the tertiary and general hospital. Carbapenem-resistance gene was detected in sewage of community tank-II and sewage from tertiary hospital. Methicillin-resistance gene was detected in sewage of community tank-II, sewage from a fish market sewage tank and sewage from an animal slaughter house sewage tank. The detection of a KPC, *van* A/B and a *mec*A in sewages will help further the process to take the appropriate measures to prevent the spread of such bacteria in the environment.

## Introduction

The wide spread use of antibiotics in human therapy, animal therapy, and live stock has resulted in the development of antibiotic-resistant isolates, leading to serious environmental and public health problems in the community [1]. In sewage water, a group of the normal flora of human gastro-intestinal tract corresponds to a large part of bacterial communities. Recently, a carbapenem-resistance gene was detected in hospital effluents in Brazil [2], municipal wastewater in Saudi Arabia [3]. *Enterobacteriaceae* with resistance to carbapenem are potentially a major global health problem [4]. Vancomycin-resistance genes (*van*A/B) and vancomycin-resistant enterococci (VRE) have been detected in sewage treatment plants in Japan [5]. In addition, VRE have also been reported from hospital sewage [6, 7], sewage of urban community, Swedish wastewater [8], and sewage from the south coast of England [9]. Methicillin-resistant *Staphylococcus aureus* (MRSA) strain was isolated from hospital effluents in India [10] and Iran [11], and *mec*A-encoded MRSA was isolated from surface water in Turkey [12]. Apart from this, use of animal feed additives such as antibiotics plays a significant role in evolving antibiotic resistance in the normal flora of animal gastrointestinal tracts [13]. Recent report states antibiotic-resistant bacteria have begun spreading into the environment and communities [14]. The prevalence of vancomycin-resistance genes, a methicillin-resistance gene and a carbapenem-resistance gene has been reported in sewages of various countries. However, to the best of our knowledge, there is no data on the prevalence of a carbapenem-resistance gene, vancomycin-resistance genes, a methicillin-resistance gene in sewages of hospitals, and municipal sewages including waste-water from the animal slaughter house and fish markets in southwestern province, Saudi Arabia. Hence, the present study was undertaken to investigate the prevalence of vancomycin-resistance genes, a methicillin-resistance gene and a carbapenem-resistance gene in the sewages of communities, sewage of hospitals, and wastewater from an animal slaughter house and a fish market by using FilmArray (BioFire Diagnostics, USA) to propose a suitable intervention to limit the spread of multidrug resistant bacteria.

## Main text

### Materials and methods

At 9 a.m. on different days, 50 mL of untreated sewage samples was collected in sterile containers (Falcon tubes; Becton–Dickinson, USA) from six sewage treatment plants: four from municipal sewage treatment plants (community sewage centre-I, community sewage centre-II, a fish market sewage tank and an animal slaughter house sewage tank) and two from hospital sewage treatment plants (a tertiary hospital sewage tank and general hospital sewage tank) in Jazan Province, Saudi Arabia. All samples were frozen [15] and transported for processing at the molecular biology laboratory at King Abdulaziz University Hospital, Jeddah. Molecular biological analysis of all the samples by FilmArray (BioFire Diagnostics, USA) was carried out within 3 days of sample collection. Multiplex PCR-based FilmArray blood culture identification panel (BCID) panel is useful to perform microbiological diagnosis directly from samples as it offers acceptable sensitivity and moderate agreement with conventional microbiological methods [16]. The FilmArray (BioFire Diagnostics, USA) BCID panel, which is an easy-to-use multiplex PCR system with a variety of diagnostic applications, was used to detect the following gram-positive bacteria, gram-negative bacteria, and yeast: Staphylococci (with a specific differentiation of *Staphylococcus aureus*); Streptococci (including specific differentiations of *Streptococcus pyogenes*, *Streptococcus pneumoniae and Streptococcus agalactiae*); Enterococci; *Listeria monocytogenes*; *Enterobacteriaceae* (with a specific differentiation of *Escherichia coli*, *Klebsiella pneumoniae*, *Klebsiella oxytoca*, *Proteus*, the *Enterobacter cloacae* complex *and Serratia marcescens*); *Acinetobacter baumannii*; *Pseudomonas aeruginosa; Haemophilus influenzae;* and *Neisseria meningitides* (encapsulated). The following antimicrobial resistance genes and their associated organisms were also detected: vancomycin-resistance genes (*vanA/B*) (Enterococci); a carbapenem-resistance gene (KPC) (any *Enterobacteriaceae*, *A. baumannii*, and *P. aeruginosa*); and a methicillin-resistance gene (*mec*A) (*Staphylococcus*). The FilmArray assay has 98% sensitivity and 99.9% specificity according to the manufacturer (BioFire Diagnostics, USA), and was approved by the United States Food and Drug Administration (FDA) for integration with molecular detection methods. A performance verification protocol was followed according to manufacturer (BioFire Diagnostics, USA). In addition, positive controls such as *K. pneumoniae* (ATCC^®^ BAA-1705), which carries the *bla*_KPC_ gene (carbapenem resistance), *E. faecalis* (ATCC^®^ 51299), which carries the *van*B gene (vancomycin resistance), *S. aureus* subsp. *aureus* (ATCC^®^ 33591), which carries the *mec*A gene (methicillin resistance), and a negative control were included along with the samples.

The reagents were placed in the FilmArray pouch loading station following the colour-coded guides. The hydration solution was injected into the pouch using a colour-coded syringe. Then, 0.1 mL of the sewage sample solution (first line of the pipette) was mixed with the sample buffer, and the sample/buffer mix was injected into the pouch using the colour-coded syringe. The pouch was then loaded into the BioFire machine. All of the sewage samples were analysed and the final results were obtained.

### Results

Vancomycin-resistance genes were detected in the sewage of the community tank-II and the sewage tank of the tertiary and general hospital. The carbapenem-resistance gene was detected in the sewage of the community tank-II and sewage from the tertiary hospital. The methicillin-resistance gene was detected in the sewage of the community tank-II, sewage from the fish market sewage tank and sewage from the animal slaughter house sewage tank (Table 1).

**Table 1:** Detection of vancomycin-resistance genes, carbapenem-resistance gene and methicillin-resistance gene and some of pathogenic gram-positive and gram-negative bacteria in various sewage samples using FilmArray

S. no	Samples from various sewage treatment plants	Antimicrobial-resistance genes			Detected bacteria	
A	Sewage from municipal sewage treatment plant, Jazan Province	*van A/B*	Carbapenem-resistance gene (KPC)	*mec*A	Gram positive-bacteria	Gram negative-bacteria
1	Sewage from community sewage center-I	Not detected	Not detected	N/A	*Enterococcus* species	*Escherichia coli*
					*Streptococcus* species	*Klebsiella pneumoniae*
2	Sewage from community sewage center-II	Detected	Detected	Detected	*Enterococcus* species	*Acinetobacter baumannii*
					*Staphylococcus aureus*	*Enterobacter cloacae* complex
					*Streptococcus agalactiae (Group B)*	*E. coli*
						*K. pneumoniae*
						*Klebsiella oxytoca*
						*Pseudomonas aeruginosa*
3	Sewage from fish market sewage tank	N/A	Not detected	Detected	*S. aureus*	*K. pneumoniae*
					*Streptococcus* species	*P. aeruginosa*
4	Sewage from animal slaughter house sewage tank	N/A	Not detected	Detected	*Staphylococcus aureus*	*E. cloacae* complex
					*Streptococcus species*	*K. pneumoniae*
B	Sewage from hospital sewage centers, Jazan Province					
1	Sewage from tertiary hospital sewage tank	Detected	Detected	N/A	*Enterococcus* species	*A. baumannii*
					*S. agalactiae (Group B)*	*E. cloacae complex*
						*E.coli*
						*K. pneumoniae*
						*K. oxytoca*
						*Serratia marcescens*
						*P. aeruginosa*
2	Sewage from general hospital sewage tank	Detected	Not detected	N/A	*Enterococcus* species	*A. baumannii*
					*S. agalactiae (Group B)*	*E. cloacae complex*
						*E.coli*
						*K. pneumoniae*
						*K. oxytoca*
						*P. aeruginosa*

*Van* A/B (vancomycin-resistance genes); carbapenem-resistance gene (KPC); *mec*A (methicillin-resistance gene)

N/A (not applied)—when no appropriate organism is detected regardless of the result for the antimicrobial resistance gene assay

*Enterococcus* species, *Streptococcus* species, *E. coli* and *K. pneumoniae* were detected in the community sewage tank-I. *Enterococcus* species, *S. aureus*, *S. agalactiae*, *A. baumannii*, the *E. cloacae* complex, *E. coli*, *K. pneumoniae*, *K. oxytoca* and *P. aeruginosa* were detected in the community sewage tank-II. *S. aureus*, *Streptococcus* species, *K. pneumoniae* and *P. aeruginosa* were detected in the sewage tank of the fish market. *S. aureus*, *Streptococcus* species, the *E. cloacae* complex and *K. pneumoniae* were detected in the sewage tank of the animal slaughter house. *Enterococcus* species, *S. agalactiae*, *A. baumannii*. The *E. cloacae* complex, *E. coli*, *K. pneumoniae*, *K. oxytoca*, *S. marcescens* and *P. aeruginosa* were detected in the sewage tank of the tertiary hospital. *Enterococcus* species, *S. agalactiae*, *A. baumannii*, *E. cloacae* complex, *E. coli*, *K. pneumoniae*, *K. oxytoca* and *P. aeruginosa* were detected in the sewage of the general hospital (Table 1).

### Discussion

The sewage from hospitals is an important hotspot for the growth and propagation of antibiotic-resistant bacteria. Spread of multidrug-resistant bacteria has become an increasing cause of concern [17]. VRE has been isolated from the sewage of communities and hospitals, and from various animals. The mechanism of vancomycin-resistant enterococci is due to the *vanA* and *vanB* cluster of genes encoding the alternative production of enterococci cell wall precursors that poorly bind vancomycin. Enterococci with acquired glycopeptide resistance have a peptidoglycan precursor end with depsipeptide d-alanyl-d-lactate, whereas normal peptidoglycan precursors end with dipeptide d-alanyl-d-alanine [18].

In the present study, vancomycin-resistance genes were detected in 1 out of the 4 sewage samples collected from municipal sewage (Table 1). However, vancomycin-resistance genes were detected in both the sewage samples from the tertiary and general hospitals. Varela et al. [6] reported the pattern of resistance to antibiotics of enterococci isolates from clinical samples and the sewage of hospitals, and similar results were found. That study also determined that the hospital sewage discharged into the urban sewage treatment plant may be the source of VRE and spread to the environment. Therefore, the sewage of hospitals may be a very important source of vancomycin-resistance genes or VRE. Another report stated that VRE may have originated from gastrointestinal colonization in patients with a prolonged stay in hospitals. The long-term use of antibiotics, prolonged stays in hospitals and severe underlying diseases have increased the risk of gastrointestinal colonization of VRE [19]. Moreover, VRE has also been detected in 12% of the sewage samples from the Miyazaki Province of Japan [7]. Vancomycin-resistance genes have been detected in a sewage treatment plant in Japan and may have been discharged into the environment in coastal areas [9]. In addition, VRE was reported from patients in hospitals in Saudi Arabia [20].

There is an increased detection of carbapenemase-producing *K. pneumoniae* in sewage and polluted waters. Montezzi et al. [21] detected carbapenemase-producing bacteria in coastal recreational waters in Brazil. In the present study, a carbapenem-resistance gene was detected in 1 out of 4 sewage samples collected from municipal sewage (Table 1.). Similarly, Mantilla-Calderon et al. [3] detected a carbapenem-resistance gene (*bla*NDM-1-positive *E. coli* strain) from municipal wastewater in Jeddah, Saudi Arabia. However, in the present study, a carbapenem-resistance gene was also detected in sewage sample from the tertiary hospital, and, similarly, a carbapenem-resistance gene was detected in hospital effluents in Brazil [2]. Carbapenem-resistant *Enterobacteriaceae* were also reported from patients in hospitals in Saudi Arabia [22]. The common cause of carbapenem-resistance is due to carbapenemase-producing *K. pneumoniae* and New Delhi metallo-β-lactamase (NDM) type β-lactamases [23].

In the present study, a methicillin-resistance gene was detected in 3 out of 4 sewage samples collected from municipal sewage, including that of an animal slaughter house and a fish market (Table 1). Similarly, Naquin et al. [24] reported a methicillin-resistance gene in both raw and treated sewage from a sewage treatment plant in Thibodaux, Louisiana, USA, and described a genetic transformation assay, which showed transformation of a methicillin-resistance gene (*mac*A) to an antibiotic-sensitive *S. aureus* that became resistant within 24 h. Meanwhile, Rahimi and Bouzari [11] reported an epidemiological connection between clinical and sewage MRSA isolates in Tehran.

In our study, vancomycin-resistance genes and a carbapenem-resistance gene were not detected in the community sewage-I sample; instead, *Enterococcus* species and *K. pneumoniae* were detected in those samples. Similarly, *Enterococcus*, vancomycin-resistance genes and a carbapenem-resistance gene were not detected in the sewage samples of the animal slaughter house and the fish market; however, a methicillin-resistance gene in *S. aureus* and *K. pneumoniae* were detected in these samples. Vancomycin-resistance genes, a carbapenem-resistance gene, a methicillin-resistance gene, *Enterococcus* species, *S. aureus* and *K. pneumoniae* were detected in the community sewage tank-II. Moreover, vancomycin-resistance genes, carbapenem-resistance genes, *Enterococcus* species and *K. pneumoniae* were detected in the sewage samples of the tertiary hospital. A carbapenem-resistance gene was not detected in the sewage of the general hospital; instead, vancomycin-resistance genes, *Enterococcus* species and *K. pneumoniae* were detected in these samples (Table 1).

The present study indicates the prevalence of vancomycin-resistance genes, a carbapenem-resistance gene and a methicillin-resistance gene in the hospital and municipal sewages in a southwestern province of Saudi Arabia that were previously not reported. Detection of more resistance genes in some of the samples may be due to the hospital sewage being discharged into the community sewage treatment plant. The United States Environmental Protection Agency and the World Health Organization advocate the use of enterococci as an indicator to check the quality of the aquatic environment [25]. Cheung et al. [25] reported the presence of enterococci in the marine water of six beaches of Hong Kong and stated that swimming in beaches contaminated with enterococci is of great concern for public health. Similarly, the presence of a carbapenem-resistance gene and a methicillin-resistance gene in the sewage represents a potential hazard to the local public health [3]. It would be better to have a sewage treatment plant in hospitals to reduce harmful impact on the environment. Moreover, studies have reported the transfer of antibiotic-resistance genes to other strains of the same bacteria or other species or genera in sewage [8]. The spread of multidrug-resistant bacteria to the environment may be due to overflow of sewage tanks and can cause a great burden if multidrug-resistant bacteria enter into the sea [26].

## Conclusions

Antimicrobial resistance gene surveillance in hospitals and municipal sewage will help efforts to better implement appropriate measures and to prevent the spread of multidrug-resistant bacteria in a geographical area.

## Limitations

In the present study prevalence of resistance genes was carried out in sewage from six sewage treatment plants: four from municipal sewage treatment plants and two from hospital sewage treatment plants in Jazan Province, Saudi Arabia. Similar study with large number of sewage samples from municipal sewage treatment plants as well as hospital sewage treatment plants will be conducted to draw final conclusion regarding spread of resistance genes in hospital and municipal sewage in a southern province of Saudi Arabia.

## Abbreviations

*van A/B*: vancomycin resistance genes A/B
KPC: carbapenem-resistance gene
*mec*A: methicillin-resistance gene
VRE: vancomycin-resistant enterococci
FDA: Food and Drug Administration
